# Eating disorder diagnostics in the digital era: validation of the Norwegian version of the Eating Disorder Assessment for DSM-5 (EDA-5)

**DOI:** 10.1186/s40337-020-00310-7

**Published:** 2020-07-09

**Authors:** Camilla Lindvall Dahlgren, B. Timothy Walsh, Karianne Vrabel, Cecilie Siegwarth, Øyvind Rø

**Affiliations:** 1 Regional Department for Eating Disorders, Division of Mental Health and Addiction, Oslo University Hospital, Ullevål HF, Postboks 4950 Nydalen, 0424 Oslo, Norway; 2 Department of Psychology, Bjørknes University College, Oslo, Norway; 3 Department of Psychiatry, College of Physicians & Surgeons, Columbia University, New York State Psychiatric Institute, New York, USA; 4 Research Institute of Modum Bad, Vikersund, Norway; 5 Institute of Clinical Medicine, University of Oslo, Oslo, Norway

**Keywords:** Eating disorders, Feeding disorders, Assessment, DSM-5, EDA-5, EDE 17.0D, Diagnosis, Interview

## Abstract

**Objective:**

The Eating Disorder Assessment for DSM-5 (EDA-5) is an electronic, semi-structured interview developed to assess feeding and eating disorders following DSM-5 criteria. The original English version has strong psychometric properties, and previous research has shown high rates of agreement between diagnoses generated by the Eating Disorder Examination (EDE) interview and the EDA-5. The current study aimed to validate the Norwegian version of the EDA-5, and is the first international validation of this diagnostic tool.

**Method:**

A total of 91 (87 females and 4 males) adult in- and out-patients were recruited from two of the largest eating disorder clinics in Norway. Diagnoses assigned using the EDA-5 were compared to diagnoses from the EDE interview (v. 17.0D).

**Results:**

Results showed that diagnoses assigned using EDE and EDA-5 were identical for 75 (82.4%) of the 91 cases. Among individual diagnostic categories, kappas ranged from moderate (.49) to perfect (1.00) agreement. The majority of discrepant cases occurred between full- and sub-threshold AN and BN. The EDA-5 was significantly quicker to administer compared to the EDE (22 vs. 54 min).

**Conclusions:**

The Norwegian EDA-5 can quickly and efficiently generate DSM-5 diagnoses without compromising diagnostic accuracy. It is a promising alternative to existing diagnostic tools, and may help streamline the identification of feeding and eating disorders in clinical settings and in research.

## Plain English Summary

This study compared the agreement between two diagnostic interviews for feeding and eating disorders; the traditional Eating Disorder Examination (EDE) and the newly developed Eating Disorder Assessment for DSM-5 (EDA-5). The EDA-5 is a web-based diagnostic instrument, and this study presents results from its first international (Norwegian) validation. We found that the EDA-5 was significantly quicker to administer compared to the EDE interview (22 vs. 54 min), and that the two instruments generated identical diagnoses in a little over 82% of the cases. It is therefore a promising alternative to traditional diagnostic interviews, which are often lengthy and require extensive training to administer.

## Background

The principle of early diagnosis and early intervention to optimize disease outcomes is widely accepted in mental health. For individuals with eating disorders, the first years of illness appears to offer a critical window for recovery, beyond which outcomes are poorer [1]. It is therefore important to quickly and accurately assess the nature of an eating disorder, so that appropriate treatment recommendations can be made and possible complications can be assessed.

In DSM-5, published in 2013, several significant changes were made to the section describing the eating disorders. The criterion requiring amenorrhea for the diagnosis of anorexia nervosa (AN) was eliminated, the minimum average frequencies of binge eating and for inappropriate compensatory behaviors were reduced from twice to once weekly in the diagnostic criteria for bulimia nervosa (BN), and binge-eating disorder (BED) was officially recognized. In addition, three disorders that were described in the section on Disorders Usually First Diagnosed in Infancy, Childhood, or Adolescence in DSM-IV were combined with the eating disorders in a section renamed Feeding and Eating Disorders in DSM-5. These disorders were pica, rumination disorder, and feeding disorder of infancy or early childhood; the last was expanded and renamed avoidant/restrictive food intake disorder (ARFID).

Since the publication of DSM-5, two of the most widely used semi-structured interview guides, the Eating Disorder Examination (EDE) v. 17.0D [2], and the Structured Clinical Interview for DSM Axis 1 Disorders DSM-5 version (SCID-5) [3], were revised to reflect the changes contained in DSM-5. Albeit being widely used, the EDE and the SCID have several limitations. The EDE v. 17.0D lacks items relevant to ARFID, pica and rumination disorder [4], and although the SCID has a module for ARFID, like the EDE, it does not assess pica or rumination disorder. In addition, although the EDE is available at no cost, individuals who wish to use the EDE are required to complete specialized training, with limited accessibility. The administration of the EDE is also time consuming, normally taking between 45 and 90 min. The SCID module for the eating disorders is brief, but is not freely available. Further, the Composite International Diagnostic Interview (CIDI) [5], a general psychiatric assessment previously used in prevalence studies [6], and was anticipated to be updated in concert with the International Classification of Diseases (ICD-11) in 2018 [7]. However, the new version is still not publicly available, and when it launches, it will no longer include a section on eating disorders (L. Chaze, personal communication, January 2017).

The Eating Disorder Assessment for DSM-5 (EDA-5) was developed to provide a guide to conducting a semi-structured interview to assess whether an individual meets criteria for an eating disorder according to DSM-5 criteria. The EDA-5 is web-based and freely available (see www.eda5.org), and was developed to minimize participant and interviewer burden [8]. It is currently the only existing semi-structured interview guide which assesses all DSM-5 feeding and eating disorders. The logic underlying the EDA-5 relies on an algorithm that selects subsequent questions based on answers already obtained, i.e., it implements diagnostic “skip rules” that avoid asking questions no longer relevant to making a diagnosis. In 2015, the validity of the EDA-5 was evaluated by comparing diagnoses generated by the EDA-5 to the results of clinical interviews and the Eating Disorder Diagnostic Scale (EDDS) [8]. High rates of agreement were observed between the EDA-5 and interviews performed by experienced clinicians [7] with kappas ranging from 0.56 (for Other Specified Feeding or Eating Disorder (OSFED)/Unspecified Feeding or Eating Disorder (UFED)) to 0.97 (for BN), supporting the validity of the EDA-5.

The aim of the current study was to test the validity of the Norwegian version of the EDA-5, following the approach of Sysko et al. [8].

## Methods

### Assessment

#### The eating disorder assessment for DSM-5 (EDA-5)

The EDA-5 [8] is an electronic, semi-structured interview assessing feeding and eating disorders in adults following DSM-5 criteria, and the frequencies of salient behavioral disturbances characteristic of these conditions (e.g., the number of objective and subjective binge eating episodes and compensatory behaviors). The following full-threshold diagnoses are assessed by the EDA-5: AN (restricting or binge-eating/purging type), BN, BED, ARFID, Pica and rumination disorder. In addition, using the EDA-5 the interviewer can assign any of the OSFED diagnoses described in DSM-5 (i.e., OSFED-AN, OSFED-BN, OSFED-BED, OSFED-Night Eating Syndrome (NES) and OSFED Purging Disorder (PD)) or UFED. As implemented, the EDA-5 relies on an algorithm that selects subsequent questions based on answers already obtained. Therefore, the number of questions administered varies across individuals, and, consequently, so does the length of administration. The English version of the EDA-5 was translated into Norwegian by a group of researchers and clinicians at Regional Department of Eating Disorders (RASP) in 2016. The translation was a close collaborative process between RASP and Eating Disorder Research Unit (EDRU) at New York State Psychiatric Institute (NYSPI), Columbia University Medical Center, with the process following World Health Organization guidelines for translation and adaptation of instruments [9]. Similar to the original interview, the Norwegian version is a computer based, electronic application (“app”), with diagnostic interview procedures and internal logic identical to those of the English version. The interview is available at no cost, and is accessible via the website www.eda5.org.

#### The eating disorder examination (EDE) v. 17.0D

The EDE [2] is a semi-structured interview generating operational ED diagnoses, as well providing measures of the range and severity of ED features. The current version of the interview (v. 17.0D) generates all DSM-5 full-, subthreshold and unspecified eating disorder diagnoses including AN, BN, BED, OSFED and UFED. The three feeding disorders Pica, ARFID and rumination disorder cannot be assessed using the EDE v. 17.0D. Four clinically derived subscales assess restraint, eating concern, shape concern and weight concern. A global severity score is calculated by averaging the four subscales. Psychometric studies show sample- and subscale dependent variations in internal consistency ranging from poor to excellent and adequate concurrent and discriminant validity [10]. The administration of the EDE requires significant training, both in the technique of interviewing, as well being familiar with the concepts and rules governing the ratings. The length of administration typically ranges from 45 to 75 min [2]. The Norwegian version of the EDE v. 17.0D was used in the current study. All EDE items were administered. DSM-5 diagnoses were assigned using the diagnostic algorithms described in the EDE interview guidelines.

### Procedure

The first author of this paper (CLD) was trained and supervised in the use of the EDA-5 by BTW, and conducted all EDA-5 interviews at RASP. The EDE interviews at RASP were conducted by two of the co-authors of this paper; a psychiatric nurse (CS) and a highly experienced clinician and senior researcher holding a PhD in psychology (ØR). CS was trained in the EDE assessment by ØR, who also supervised CS throughout the data collection period. CLD trained the head of research and clinical psychologist (KV) and colleagues in the use of the EDA-5 at Modum Bad. All participating staff at Modum Bad had prior experience and formal training in the use of the EDE interview.

Participants were individuals receiving treatment at one of the two Norwegian tertiary care centers: RASP at Oslo University Hospital, Oslo, or the Eating Disorder Clinic at Modum Bad Psychiatric Center in Vikersund. At RASP, participants were recruited from the outpatient clinic and two inpatient adult clinics. A team of four psychologists, one psychiatrist, two medical doctors and two psychiatric nurses completed the EDE and the EDA-5 interviews at Modum Bad. All participants were inpatients. The order of the two interviews was counterbalanced, and intended to occur no more than five days a part (average time between the interviews = 1.1 ± 1.8 days; range 0–10 days), and conducted by different interviewers to avoid contamination. All interviews were conducted in-person. Interviewers recorded the length of the interviews and completed a checklist where fulfilled diagnostic criteria and assigned DSM-5 diagnoses were registered. Inclusion criteria were liberal, with patients being considered eligible if they were medically stable, aged 16 years or above, and provided written consent to participate in the study. No remuneration was offered. The study was approved by the Norwegian Regional Committee for Medical and Health Research Ethics (ref. 2017/8130) and the Norwegian Data Protection Authority at Oslo University Hospital. Diagnostic data was collected at the two clinics between November 2017 and June 2019.

### Statistical analyses

All statistical analyses were carried out using IBM SPSS Statistics Version 25. The sample size (*n* = 91) was modelled on the original validation study [8]. The EDE was used as a reference instrument in all analyses comparing diagnoses. Since the EDE is not designed to assign pica, rumination disorder or ARFID, reliability rates were not calculated for these three diagnoses. Kappas, sensitivity, specificity, negative and positive predictive value and accuracy was calculated for all assigned diagnoses. The five latter measures are expressed in percentages. Kappas (ranging from − 1 to + 1) were used to measure diagnostic agreement. A slightly less liberal kappa standard was applied than that reported in the original EDA-5 validation study (i.e. [11, 12]), with kappa coefficients (κ) being interpreted as follows κ < 0 = “No Agreement”, .0–.20 = “None”, .21–.39 = “Minimal”, .40–.59 = “Weak”, .60–.79 = “Moderate”, .80–.90 = “Strong” and > .90 = “Almost perfect” [13]. According to McHugh [14], any kappa above 0.60 indicates acceptable agreement among raters, whereas little confidence should be placed in results showing kappas below 0.6. In this study, sensitivity (true positives), is defined as the proportion of individuals with a specific EDE diagnosis who were accurately (i.e., identically) diagnosed using the EDA-5. Specificity (true negatives) is the proportion of individuals who did *not* receive a specific EDE diagnosis, who also did *not* receive that particular diagnosis using the EDA-5. The positive predictive value (PPV) is the probability that an individual diagnosed using the EDA-5 received that diagnosis using the EDE. The negative predictive value (NPV) is the probability that an individual, who did not receive a certain diagnosis using the EDA-5, did not receive that diagnosis via the EDE. The closer PPV and NPV values are to 1.0 (i.e. 100%), the higher the probability that the instrument being validated (in this case, the EDA-5) is doing as good as “gold standard” (in this case, the EDE interview) [14]. Accuracy is the proportion of true results, either true positives or true negatives. It is calculated as the sum true positives and true negatives divided by the sample size (n). Since no OSFED-BED diagnoses were assigned, sensitivity, negative predictive value or accuracy could not be calculated for these diagnoses.

## Results

A total of 101 adult participants were recruited to take part in the study, 57 from RASP and 44 from Modum Bad. Seven of the participants from RASP withdrew before having entered the study, and at Modum Bad, three participants were excluded due to interviews taking place too far apart (2–6 months). The final sample consisted of a total of 91 (87 females and 4 males) participants. Demographic characteristics and assigned EDE diagnoses are presented in Table 1. Neither the EDE nor the EDA-5 identified cases of OSFED-BED, OSFED-PD, OSFED-NES or UFED diagnoses. Similarly, none of the participants received a diagnosis of ARFID, pica or rumination disorder. The label “OSFED Other” was used to group participants whose eating disorder symptoms strayed too far from the individual OSFED categories, and deviated significantly from the examples given in the DSM-5. Site differences were observed for age [*F* (2, 89) = .46, *p* = .01] (patients recruited from Modum Bad were significantly older), but not for gender or BMI. The EDA-5 was significantly quicker to administer (Mean = 21.6 min, *SD* = 8.5) compared to the EDE interview (Mean = 54.0 min, *SD* = 22.1), t (79) = 12.9, *p* < .0005 (two-tailed). There were no significant differences between sites in the time required to conduct the EDE and EDA-5 interviews.

**Table 1:** Demographic characteristics and EDE diagnoses in the full sample (*n* = 91) and across sites

	Full sample (*N* = 91)	RASP (*n* = 50)	Modum Bad (*n* = 41)
Age (years), mean (*SD*), range	30.9 (9.8) 17–56	28.5 (8.8) 17–52	33.8 (10.2)^**^ 19–56
Body mass index mean (*SD*), range	21.6 (7.9) 12.5–51.4	22.4 (9.2) 12.5–51.4	20.6 (5.8) 13.3–39.2
Female, n (%)	87 (95.6%)	48 (96.0%)	39 (95.1%)
Diagnosis (EDE), n (%)			
AN	34 (37.4%)	15 (30.0%)	19 (46.4%)
*AN-R*	16 (17.6%)	7 (14.0%)	9 (22.0%)
*AN-BP*	19 (19.8%)	8 (16%)	10 (24.4%)
BN	25 (27.5%)	11 (22.0%)	14 (34.1%)
BED	6 (6.6%)	5 (10.0%)	1 (2.4%)
OSFED	25 (27.5%)	18 (36.0%)	7 (17.1%)
*OSFED-AN*	14 (15.4%)	10 (20.0%)	4 (9.8%)
*OSFED-BN*	4 (4.4%)	3 (6.0%)	1 (2.4%)
*OSFED-Other*	7 (7.7%)	5 (10.0%)	2 (4.9%)
No ED	1 (1.1%)	1 (2.0%)	0 (0%)

*EDE* The Eating Disorder Examination v. 17.0D, *AN* Anorexia Nervosa, *AN-R* Anorexia Nervosa Restrictive type, *AN-BP* Anorexia Nervosa Binge-eating/Purging type, *BN* Bulimia Nervosa, *BED* Binge Eating Disorder, *OSFED* Other Specified Feeding and Eating Disorders, *ED* Eating Disorder, *RASP* Regional Department for Eating Disorders. ^**^ = Patients recruited from Modum Bad were significantly older than patients recruited from RASP (*p* ≤ .01)

### Interview discrepancies

Diagnostic distribution using the EDE and the EDA-5 is presented in Table 2. Diagnoses assigned using EDE and EDA-5 interviews were identical for 75 (82.4%) of the 91 cases. Among individual diagnostic categories, kappas ranged from moderate (.49) to perfect (1.00) agreement. Kappas, sensitivity, specificity, positive and negative predictive values as well as agreement accuracy are presented in Table 3.

**Table 2:** Diagnostic distribution in the full sample (*n* = 91) using the EDE and the EDA-5

Diagnosis (%, n)	EDE	EDA-5
AN total	36.2% (33)	51.7% (47)
AN-R	16.5% (15)	23.1% (21)
AN-BP	19.8% (18)	28.6% (26)
BN	27.5% (25)	20.9% (19)
BED	6.6% (6)	6.6% (6)
OSFED total	27.5% (25)	19.8% (18)
OSFED-AN	15.4% (14)	5.5% (5)
OSFED-BN	4.4% (4)	4.4% (4)
OSFED-BED	0% (0)	0% (0)
OSFED-PD	0% (0)	0% (0)
OSFED-NES	0% (0)	0% (0)
OSFED-Other	7.7% (7)	9.9% (9)
ARFID	NA	0% (0)
Pica	NA	0% (0)
Rumination disorder	NA	0% (0)
No ED	1.1% (1)	1 (1.1%)

*EDE* The Eating Disorder Examination v. 17.0D, *EDA-5* The Eating Disorder Assessment for DSM-5, *AN* Anorexia Nervosa, *AN-R* Restrictive type, *AN-BP* Binge-eating/Purging type, *BN* Bulimia Nervosa, *BED* Binge Eating Disorder, *OSFED* Other Specified Feeding and Eating Disorders, *OSFED-PD* Purging Disorder, *OSFED-NES* Night Eating Syndrome, *ARFID* Avoidant Restrictive Food Intake Disorder, *ED* Eating Disorder, AN total includes AN-R and AN-BP, OSFED total includes OSFED-AN, OSFED-BN, OSFED-BED, OSFED-PD and OSFED-NES

**Table 3:** Agreement of the EDA-5 with the EDE interview (*n* = 91)

Diagnosis	*κ*	Sensitivity	Specificity	Positive predictive value	Negative predictive value	Accuracy
**EDE**^a^						
AN total	.72^**^	1.00	.77	1.00	.72	.85
AN-R	.77^**^	.94	.92	.99	.73	.93
AN-BP	.79^**^	1.00	.90	1.00	.72	.91
BN	.82^**^	.76	1.00	.92	1.00	1.00
BED	1.00^**^	1.00	1.00	1.00	1.00	1.00
OSFED total	.62^**^	.50	1.00	.89	1.00	1.00
OSFED-AN	.53^**^	.40	1.00	.89	1.00	1.00
OSFED-BN	1.00^**^	1.00	1.00	1.00	1.00	1.00
OSFED Other	.86^**^	1.00	.98	1.00	.78	.98
No diagnosis	.49^**^	.33	1.00	.98	1.00	1.0

*EDA-5* Eating Disorder Assessment for DSM-5, *EDE* Eating Disorder Examination, *AN* Anorexia Nervosa, *AN-R* Restrictive type, *AN-BP* Binge-eating/Purging type, *BN* Bulimia Nervosa, *BED* Binge Eating Disorder, *OSFED* Other Specified Feeding and Eating Disorders. AN total includes AN-R and AN-BP, OSFED total includes OSFED-AN, and OSFED-BN. No OSFED-BED, PD or NES cases were diagnosed using the EDE and the EDA-5. ^a^EDE is used as the reference assessment in all analyses. **p < .0005

When comparing diagnoses assessed using the EDA-5 and the EDE interview, 16 out of 91 patients (17.6%) received discrepant diagnoses (see Table 4). Twelve of these were patients at RASP, and the remaining four were patients at Modum Bad.

**Table 4:** Diagnoses assigned using the EDA-5 and the EDE (*n* = 91)

		EDA-5							
		AN-R	AN-BP	BN	BED	OSFED-AN	OSFED-BN	UFED	No FED
**EDE**	AN-R	**15**	1	0	0	0	0	0	0
	AN-BP	0	**18**	0	0	0	0	0	0
	BN	0	5	**19**	0	0	0	1	0
	BED	0	0	0	**6**	0	0	0	0
	OSFED-AN	6	2	0	0	**5**	0	1	0
	OSFED-BN	0	0	0	0	0	**4**	0	0
	OSFED Other	0	0	0	0	0	0	**7**	0
	No FED	0	0	0	0	0	0	0	**1**

*EDA-5* Eating Disorder Assessment for DSM-5, *EDE* Eating Disorder Examination, *AN* Anorexia Nervosa, *AN-R* Restrictive type, *AN-BP* Binge-eating/Purging type, *BN* Bulimia Nervosa, *BED* Binge Eating Disorder, *OSFED* Other Specified Feeding or Eating Disorder, *FED* Feeding and Eating Disorder. **Bold** figures indicate diagnostic *agreement* between the EDA-5 and the EDE

## Discussion

This study examined the validity of the Norwegian version of the web-based diagnostic tool, the EDA-5, in assigning DSM-5 feeding and eating disorders. In line with the original study [8], the Norwegian EDA-5 quickly and efficiently generated DSM-5 diagnoses without compromising diagnostic accuracy. As such, it is a promising alternative to existing diagnostic tools, and may facilitate the identification of eating disorders in clinical settings as well as in research.

Similar to the sample in the original study [8] the majority of participants in the present study were adult Caucasian females presenting with BMIs ranging from 13 to 51 kg/m^2^. Also, similar to Sysko et al. [8] the highest level of agreement between the EDA-5 and the EDE (1.00) was found for BN and the lowest kappa (0.62), sensitivity (0.50) and PPV (0.89) were found for the OSFED group. The only two diagnostic categories where there were no discrepancies at all were BED and OSFED-BN, partially (due to low N), and possibly underscoring the clarity of the criteria for these categories. Considering the relatively new inclusion of BED as a separate diagnostic entity, these are promising results. The majority of discrepant cases occurred between full- and sub-threshold AN and BN, likely reflecting different time frames used by the interviews in assessing DSM-5 diagnostic criteria A (“*Restriction of energy intake relative to requirements leading to a significantly low body weight”)*. DSM-5 does not explicitly specify a time frame over which weight should be assessed; the EDA-5 aims to determine whether an individual has been at a significantly low body weight over the last three months, whereas the EDE focuses only on the *current* weight. In 12 of the 16 discrepant cases, the individual had been at a significantly low weight during the previous three months but was not significantly underweight at the time of the interview. If the EDA-5 had focused on *current* weight, or if the EDE (or the clinician doing the EDE) had prompted for lowest weight the previous three months, diagnostic agreement would have risen to 95.6%. These numbers are in contrast to those in the measures’ original validation [8], suggesting that how weight is judged in practice impacts diagnostic distributions and rates. Whereas BN and BED diagnoses require a three-month minimum duration of binge eating (for BN and BED) and compensatory behaviors (for BN only), the DSM-5 does not specify the amount of time an individual should have been at a normal weight to be considered recovered from AN [10]. Nor does it specify the amount of time an individual should have been underweight to be considered fulfilling the AN weight criterium. The EDA-5, on the other hand, has implemented the same 3-month time frame as required for full threshold BN and BED. The rationale for not simply using the day-of-evaluation weight is that weight can fluctuate greatly over short periods of time, especially in individuals enrolled in nutrition restoration regimens, including inpatient treatments for AN. Judging the AN weight criteria over the same duration used to judge the frequencies of behavioral disturbances in BN and BED, will presumably decrease diagnostic crossover and reduce the number of OSFED cases, the latter being one of the top priorities of the revisions implemented in DSM-5 [6].

In one (case #1) of the four remaining discrepant cases, the participant fulfilled BN criteria B through E, but reported objective binge eating *without* loss of control and was assigned an OSFED Other diagnosis in the EDA-5. In the EDE, the same patient fulfilled AN criteria B and C (but not A since she was normal weight), with *subjective* binge eating and received an OSFED-AN diagnosis. One participant (case #2) received a BN diagnosis using the EDE, and an AN-BP diagnosis using the EDA-5; the interviewing clinician using the EDE interview assessed her weight (and BMI which was 18.0) as not being “significantly low” and therefore not fulfilling the AN weight criterion. The interviewing EDA-5 clinician, on the other hand, assessed the patient’s weight/BMI as being significantly low, fulfilling criteria for an AN diagnosis. One participant (case #3) received an AN-R diagnosis using the EDE, and an AN-BP diagnosis using the EDA-5; the use of laxatives was interpreted as a compensatory behavior in the EDA-5 but not in the EDE. The final (case #4) discrepant case occurred as one participant fulfilled all but one BN criterion (D; self-evaluation is unduly influenced by body shape and weight) and received an OSFED Other diagnosis when assessed using the EDA-5; when using the EDE, the clinician judged that criterion D was met, and assigned the patient a BN diagnosis. Discordant diagnoses, such as the four cases described above, can be explained by differences in clinical judgment (e.g. case #3), different interpretations of diagnostic criteria (e.g. case #2 and #4) or by different patient reports (e.g. case #1). These are all important factors to consider in the diagnostic process, whether it is for research or clinical purposes.

### Strengths and limitations

Strengths of this study include standardized procedural steps for translation and validation, and data collection at two different sites. Also, high rates of diagnostic agreement were reached despite variability in interviewers’ professional degree and specialty, supporting the utility of the EDA-5 across professions and experience with feeding and eating disorders. In addition, minimal resources were required to train interviewers to use of EDA-5. Neither the current study nor that of Sysko et al. assessed individuals with ARFID, pica, or rumination disorder, so the validity of the EDA-5 in characterizing those disorders is unknown. In addition, more research is needed to investigate the applicability of the EDA-5 in younger populations, and to determine the extent to which these results generalize to males and ethnically diverse samples. Replication in larger samples of OSFED (e.g. in non-clinical samples) would be beneficial to assess the diagnostic reliability of the assessment of OSFED subthreshold conditions. This may require more detailed criteria to enhance diagnostic concordance within and between measures. Methodological limitations such as the lack of inter-rater and test-retest reliability should also be noted.

## Conclusion

The EDA-5 is currently the only available semi-structured interview capable of assessing all the feeding and eating disorders described in DSM-5. The current study replicates and extends the report of Sysko et al. [8] in documenting that, even after translation to Norwegian and in the hands of clinicians who were not involved in its development, the EDA-5 efficiently provides valid diagnostic assessments of eating disorders following DSM-5 criteria. Although they have not been formally tested, versions of the EDA-5 in Spanish and Turkish are also available (see www.eda5.org).

## Data Availability

The datasets used and/or analysed during the current study are available from the corresponding author on reasonable request.

## Abbreviations

AN: Anorexia Nervosa
AN-R: Anorexia Nervosa Restrictive subtype
AN-BP: Anorexia Nervosa Binge-Purge subtype
ARFID: Avoidant/Restrictive Food Intake Disorder
BED: Binge Eating Disorder
BN: Bulimia Nervosa
CIDI: Composite International Diagnostic Interview
DSM: Diagnostic and Statistical Manual of Mental Disorders
ED: Eating Disorder
EDA-5: Eating Disorder Assessment for DSM-5
EDDS: Eating Disorder Diagnostic Scale
EDE: The Eating Disorder Examination
EDRU: Eating Disorder Research Unit
ICD: International Classification of Diseases
NES: Night Eating Syndrome
NPV: Negative Predictive Value
NYSPI: New York State Psychiatric Institute
OSFED: Other Specified Feeding and Eating Disorders
PD: Purging Disorder
PPV: Positive Predictive Value
RASP: Regional Department for Eating Disorders
SCID: Structured Clinical Interview for DSM Axis 1 Disorders
UFED: Unspecified Feeding or Eating Disorders
